# Crystal structure of SrGeO_3_ in the high-pressure perovskite-type phase

**DOI:** 10.1107/S2056989015007264

**Published:** 2015-04-18

**Authors:** Akihiko Nakatsuka, Hiroshi Arima, Osamu Ohtaka, Keiko Fujiwara, Akira Yoshiasa

**Affiliations:** a Graduate School of Science and Engineering, Yamaguchi University, Ube 755-8611, Japan; b Institute for Materials Research, Tohoku University, Sendai 980-8577, Japan; c Graduate School of Science, Osaka University, Toyonaka 560-0043, Japan; d Graduate School of Science and Technology, Kumamoto University, Kumamoto 860-8555, Japan

**Keywords:** crystal structure, strontium germanate, perovskite, high-pressure phase

## Abstract

Single crystals of the SrGeO_3_ (strontium germanium trioxide) high-pressure phase have been synthesized successfully at 6 GPa and 1223 K. The compound crystallizes with the ideal cubic perovskite-type structure (space group *Pm*-3*m*), which consists of a network of corner-linked regular GeO_6_ octa­hedra (point-group symmetry *m*-3*m*), with the larger Sr atoms located at the centers of cavities in the form of SrO_12_ cubocta­hedra (point-group symmetry *m*-3*m*) in the network. The degrees of covalencies included in the Sr—O and the Ge—O bonds calculated from bond valences are 20.4 and 48.9%, respectively. Thus, the Ge—O bond of the GeO_6_ octa­hedron in the SrGeO_3_ perovskite has a strong covalency, comparable to those of the Si—O bonds of the SiO_4_ tetra­hedra in silicates with about 50% covalency. The thermal vibrations of the O atoms in the title compound are remarkably suppressed in the directions of the Ge—O bonds. This anisotropy ranks among the largest observed in stoichiometric cubic perovskites.

## Abstract 2

The high-pressure phase of SrGeO_3_ synthesized at 6 GPa and 1223 K adopts the ideal cubic perovskite-type structure. The Ge—O bond is largely covalent, which influences the thermal vibration behavior of the O atom.

## Chemical context   

The phase transitions of the perovskite-type compounds *AB*O_3_ have long attracted much attention for various industrial applications, as represented in ferroelectric substances such as BaTiO_3_. The strontium germanate SrGeO_3_ undergoes a sequence of phase transitions at high pressures and high temperatures of pyroxenoid (pseudowollastonite) type → walstromite type → perovskite type (Shimizu *et al.*, 1970 ▸; Akaogi *et al.*, 2005 ▸). In a recent study (Mizoguchi *et al.*, 2011 ▸), it was reported that the high-pressure perovskite-type phase of SrGeO_3_ is a promising transparent electronic conductor. A detailed structural study of this perovskite-type phase is important to elucidate the origin of the conduction mechanism. Despite such importance, the high-pressure perovskite-type phase has been studied so far only on the basis of polycrystalline samples and its powder X-ray diffraction pattern has only suggested that it adopts the ideal cubic perovskite structure. Perovskite-type compounds are well-known to have various symmetries owing to a slight tilting of the *B*O_6_ octa­hedra (Glazer, 1972 ▸, 1975 ▸). However, it is often difficult to determine their actual symmetries from powder X-ray diffraction techniques. Thus, more precise data based on single crystal X-ray diffraction are indispensable for the determination of the crystal structure of the SrGeO_3_ high-pressure perovskite-type phase. We recently succeeded in the growth of SrGeO_3_ perovskite-type single crystals at high pressure and high temperature. The crystal structure refined from single-crystal X-ray diffraction data is reported here.

## Structural commentary   

**Figure 1:**
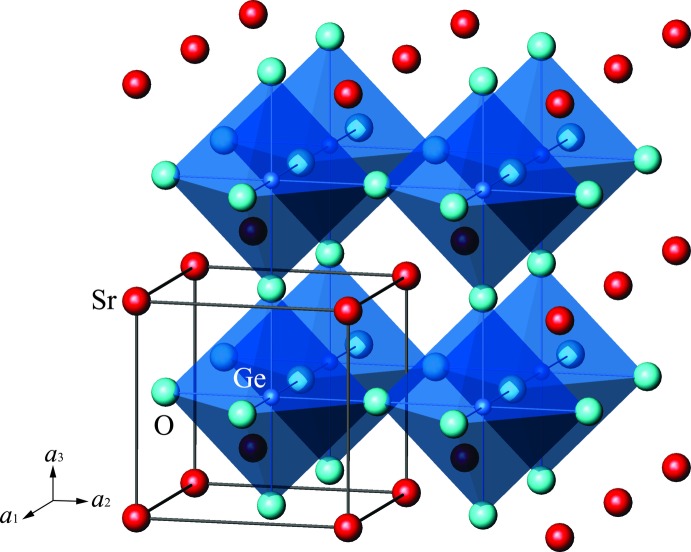
Representation of the SrGeO_3_ perovskite-type structure showing corner-linked GeO_6_ octa­hedra.

The high-pressure phase of SrGeO_3_ crystallizes with the cubic perovskite-type structure (space group *Pm* 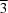 *m*). The crystal structure consists of a network of corner-linked regular GeO_6_ octa­hedra with the larger Sr atoms located at the centers of cavities in the network, forming SrO_12_ cubocta­hedra (Fig. 1 ▸). As a result of the ideal symmetry, tilts and distortions of the GeO_6_ octa­hedra are not present. The Sr, Ge and O atoms occupy Wyckoff positions 1*a* (0, 0, 0), 1*b* (0.5, 0.5, 0.5) and 3*c* (0, 0.5, 0.5), respectively, without any freedom of atomic positions. The corresponding site symmetries are *m* 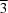 *m*, *m* 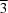 *m* and 4/*mm.m*, respectively. The observed Sr—O distance in the SrO_12_ cubocta­hedron and the Ge—O distance in the GeO_6_ octa­hedron are 2.6855 (1) Å and 1.8989 (1) Å, respectively, which are much shorter than the distances expected from the effective ionic radii (Sr—O = 2.84 Å, Ge—O = 1.93 Å; Shannon, 1976 ▸). The ratios of covalency included in the bonds calculated from *f*′_c_/*s* (= *as* ^M-1^) are 20.4% for the Sr—O bond and 48.9% for the Ge—O bond, where *f*′_c_, given by *as* ^M^ (Brown & Shannon, 1973 ▸), is the covalence in bonds; *s* is the bond valence; *a* and M are parameters relating covalence to bond valence. This value of the present Ge—O bond ranks among the largest in *B*—O bonds of *B*O_6_ octa­hedra in *A* ^2+^ *B* ^4+^O_3_-type cubic perovskites (*A* = twelvefold-coordinated cations, *B* = sixfold-coordinated cations) [*cf.* 39.8% for the Ti—O bond in SrTiO_3_ (Abramov *et al.*, 1995 ▸) and 37.8% for the Zr—O bond in BaZrO_3_ (Levin *et al.*, 2003 ▸)]. It is noteworthy, thus, that the Ge—O bond of the GeO_6_ octa­hedron in the present crystal has a strong covalency comparable to those of the Si—O bonds of the SiO_4_ tetra­hedra in silicates with about 50% covalency.

**Figure 2:**
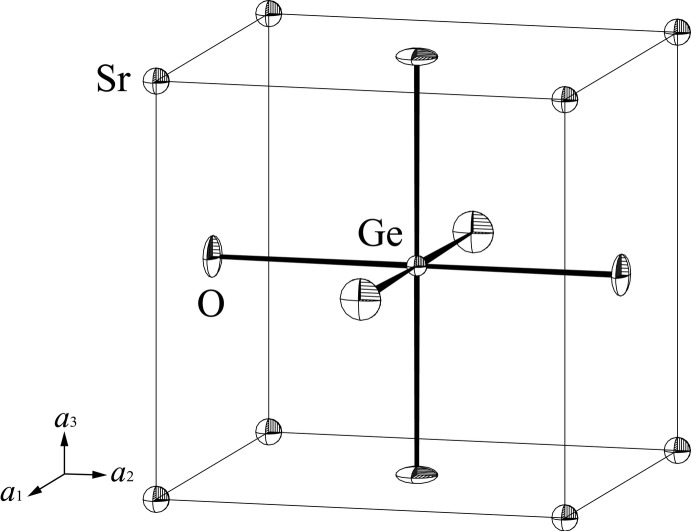
The unit cell of the cubic SrGeO_3_ perovskite with displacement ellipsoids drawn at the 80% probability level.

The site-symmetry constraints require that the displacement ellipsoids of the Sr and Ge atoms are always spherical and that of the O atom is an uniaxial ellipsoid with one determinable ellipsoid-axis in the direction of the Ge—O bond and two undeterminable ones in the directions perpendicular to it. The mean-square displacement (MSD) of the O atom is the smallest [〈*u*_S_^2^〉 = 0.0011 (8) Å^2^] in the former direction and the largest [〈*u*_L_^2^〉 = 0.0077 (7) Å^2^] in the latter directions. The 〈*u*_S_^2^〉/〈*u*_L_^2^〉 ratio of 0.14 calculated for the present crystal indicates that the displacement ellipsoid of the O atom is remarkably compressed in the former directions, as shown in Fig. 2 ▸. Such remarkable anisotropy is commonly observed in cubic perovskites with stoichiometric compositions, and the present 〈*u*_S_^2^〉/〈*u*_L_^2^〉 ratio ranks among the smallest observed [*cf.* 〈*u*_S_^2^〉/〈*u*_L_^2^〉 = 0.14 for LaAlO_3_ (Nakatsuka *et al.*, 2005 ▸), 0.43 for SrTiO_3_ (Abramov *et al.*, 1995 ▸), 0.38 for KTaO_3_ (Zhurova *et al.*, 2000 ▸), 0.50 for SrFeO_3_ (Hodges *et al.*, 2000 ▸) and 0.29 for BaZrO_3_ (Levin *et al.*, 2003 ▸)]. The remarkable anisotropy of the MSD of the O atom in the SrGeO_3_ perovskite-type structure might be related to the strong covalency of the Ge—O bond.

## Synthesis and crystallization   

**Figure 3:**
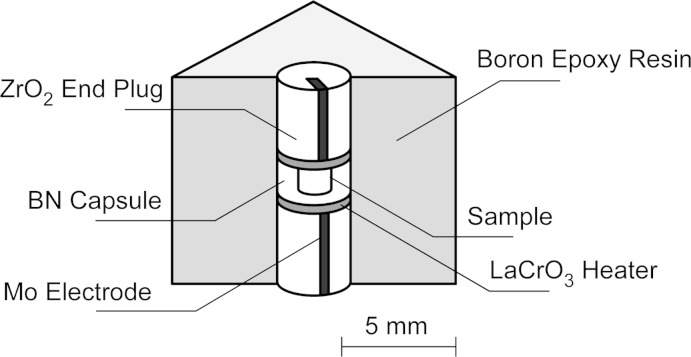
Cell assembly used in the synthetic experiment at high pressure.

A polycrystalline sample of SrGeO_3_ pseudowollastonite as the starting material was prepared by solid-state reaction of special grade reagents SrCO_3_ and GeO_2_. The resulting SrGeO_3_ pseudowollastonite material was charged in a gold capsule and then put into a BN sample chamber. As shown in Fig. 3 ▸, the sample chamber was put between a pair of LaCrO_3_ disc heaters and encased in a cubic-shaped pressure-transmitting medium made of boron-ep­oxy resin. This cell assembly was compressed with a 700 ton cubic anvil-type press. After being kept at 6 GPa and 1223 K for 1 h, the product was quenched by shutting off the electric power supply. The pressure was then released slowly and the product was recovered at ambient conditions. Single crystals of SrGeO_3_ perovskite were found in the recovered sample, together with an unknown single-crystal phase.

## Refinement   

The unit-cell parameters of the crystal under investigation assuming a triclinic cell only exhibit a minute deviation from a cubic unit cell [*a =* 3.7979 (2), *b =* 3.7978 (3), *c* = 3.7972 (3) Å, *α* = 89.984 (6), *β* = 89.997 (6), *γ* = 89.988 (5)°]. Systematic absences of reflections also agreed with space group *Pm* 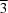 *m*. Indeed, the present crystal was satisfactorily refined in the ideal cubic perovskite structure as judged from the excellent reliability indices (Table 1 ▸).

Intensity data were averaged in Laue symmetry *m* 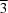 *m* to give 116 independent reflections. Of these, independent reflections with *F*_o_ 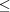 3σ(*F*_o_) were omitted for refinement. Even if independent reflections had intensities of *F*_o_ > 3σ(*F*_o_) after averaging, those averaged from a data set of equivalent reflections including reflection(s) with *F*_o_ 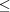 3σ(*F*_o_) were also discarded since these reflections were potentially affected by multiple diffraction. Moreover, independent reflections with (sin θ)/λ < 0.220 Å^−1^ were eliminated to reduce secondary extinction effects and to avoid dependence on atomic charge as far as possible in the choice of atomic scattering factors. Finally, 64 independent reflections were used in the present refinement. Several correction models for the secondary extinction effects were attempted during the refinement, and the isotropic correction of Type I (Becker & Coppens, 1974*a* ▸,*b* ▸) with a Gaussian mosaic spread distribution model yielded the best fits. Crystal data, data collection and structure refinement details are summarized in Table 1 ▸.

## Supplementary Material

Crystal structure: contains datablock(s) General, I. DOI: 10.1107/S2056989015007264/wm5141sup1.cif

Structure factors: contains datablock(s) I. DOI: 10.1107/S2056989015007264/wm5141Isup2.hkl

CCDC reference: 1059192

Additional supporting information:  crystallographic information; 3D view; checkCIF report

## Figures and Tables

**Table 1:** Experimental details

Crystal data	
Chemical formula	SrGeO_3_
*M*_r_	208.23
Crystal system, space group	Cubic, *P* *m* 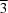 *m*
Temperature (K)	296
*a* ()	3.7978(2)
*V* (^3^)	54.78(1)
*Z*	1
Radiation type	Mo *K*
(mm^1^)	37.73
Crystal size (mm)	0.10 0.08 0.08

Data collection	
Diffractometer	Rigaku AFC-7R
Absorption correction	scan (North *et al.*, 1968 ▸)
*T*_min_, *T*_max_	0.037, 0.049
No. of measured, independent and observed [*F* > 3(*F*)] reflections	521, 116, 66
*R*_int_	0.023
(sin /)_max_ (^1^)	1.219

Refinement	
*R*[*F* > 3(*F*)], *wR*(*F*), *S*	0.011, 0.010, 1.95
No. of reflections	64
No. of parameters	6
_max_, _min_ (e ^3^)	1.04, 1.38

Computer programs: *WinAFC* (Rigaku, 1999 ▸), *RADY* (Sasaki, 1987 ▸), *ATOMS for Windows* (Dowty, 2000 ▸) and *publCIF* (Westrip, 2010 ▸).

## supplementary crystallographic information

### Crystal data

**Table:**

SrGeO_3_	*D*_x_ = 6.315 Mg m^−^^3^
*M**_r_* = 208.23	Mo *K*α radiation, λ = 0.71069 Å
Cubic, *P**m*3*m*	Cell parameters from 25 reflections
Hall symbol: -P 4 2 3	θ = 21.5–25.0°
*a* = 3.7978 (2) Å	µ = 37.73 mm^−^^1^
*V* = 54.78 (1) Å^3^	*T* = 296 K
*Z* = 1	Fragment, colorless
*F*(000) = 94	0.10 × 0.08 × 0.08 mm

### Data collection

**Table:**

Rigaku AFC-7R diffractometer	*R*_int_ = 0.023
ω–2θ scans	θ_max_ = 60.0°
Absorption correction: ψ scan (North *et al.*, 1968)	*h* = 0→9
*T*_min_ = 0.037, *T*_max_ = 0.049	*k* = 0→9
521 measured reflections	*l* = 0→9
116 independent reflections	3 standard reflections every 150 reflections
66 reflections with *F* > 3.0σ(*F*)	intensity decay: none

### Refinement

**Table:**

Refinement on *F*	Weighting scheme based on measured s.u.'s *w* = 1/σ^2^(*F*)
*R*[*F*^2^ > 2σ(*F*^2^)] = 0.019	(Δ/σ)_max_ < 0.001
*wR*(*F*^2^) = 0.020	Δρ_max_ = 1.04 e Å^−^^3^
*S* = 1.95	Δρ_min_ = −1.38 e Å^−^^3^
64 reflections	Extinction correction: isotropic Type I (Becker & Coppens, 1974*a*,*b*)
6 parameters	Extinction coefficient: 0.40 (1)E3

### Fractional atomic coordinates and isotropic or equivalent isotropic displacement parameters (Å^2^)

**Table:**

	*x*	*y*	*z*	*U*_iso_*/*U*_eq_	
Sr	0.0000	0.0000	0.0000	0.00300 (9)	
Ge	0.5000	0.5000	0.5000	0.00182 (9)	
O	0.0000	0.5000	0.5000	0.0055 (7)	

### Atomic displacement parameters (Å^2^)

**Table:**

	*U*^11^	*U*^22^	*U*^33^	*U*^12^	*U*^13^	*U*^23^
Sr	0.0030 (2)	0.0030	0.0030	0.0000	0.0000	0.0000
Ge	0.0018 (2)	0.0018	0.0018	0.0000	0.0000	0.0000
O	0.0011 (8)	0.0077 (7)	0.0077	0.0000	0.0000	0.0000

### Geometric parameters (Å, º)

**Table:**

Sr—O	2.6855 (1)	Ge—O^i^	1.8989 (1)
Sr—O^i^	2.6855 (1)	Ge—O^ii^	1.8989 (1)
Sr—O^ii^	2.6855 (1)	Ge—O^xii^	1.8989 (1)
Sr—O^iii^	2.6855 (1)	Ge—O^xiii^	1.8989 (1)
Sr—O^iv^	2.6855 (1)	Ge—O^xiv^	1.8989 (1)
Sr—O^v^	2.6855 (1)	O—O^i^	2.6855 (1)
Sr—O^vi^	2.6855 (1)	O—O^ii^	2.6855 (1)
Sr—O^vii^	2.6855 (1)	O—O^v^	2.6855 (1)
Sr—O^viii^	2.6855 (1)	O—O^vi^	2.6855 (1)
Sr—O^ix^	2.6855 (1)	O—O^xv^	2.6855 (1)
Sr—O^x^	2.6855 (1)	O—O^xvi^	2.6855 (1)
Sr—O^xi^	2.6855 (1)	O—O^xii^	2.6855 (1)
Ge—O	1.8989 (1)	O—O^xiii^	2.6855 (1)
			
O—Sr—O^i^	60.00	Sr—O—O^vi^	60.00
O—Sr—O^ii^	60.00	Sr—O—O^xv^	120.00
O—Sr—O^iii^	120.00	Sr—O—O^xvi^	120.00
O—Sr—O^iv^	120.00	Sr—O—O^xii^	120.00
O—Sr—O^v^	60.00	Sr—O—O^xiii^	120.00
O—Sr—O^vi^	60.00	Ge—O—Ge^xx^	180.00
O—Sr—O^vii^	180.00	Ge—O—O^i^	45.00
O—Sr—O^viii^	90.00	Ge—O—O^ii^	45.00
O—Sr—O^ix^	120.00	Ge—O—O^v^	135.00
O—Sr—O^x^	90.00	Ge—O—O^vi^	135.00
O—Sr—O^xi^	120.00	Ge—O—O^xv^	135.00
O—Sr^i^—O^ii^	60.00	Ge—O—O^xvi^	135.00
O—Sr^i^—O^iii^	120.00	Ge—O—O^xii^	45.00
O—Sr^ii^—O^iv^	120.00	Ge—O—O^xiii^	45.00
O—Ge—O^i^	90.00	Ge—O^xx^—O^v^	45.00
O—Ge—O^ii^	90.00	Ge—O^xx^—O^vi^	45.00
O—Ge—O^xii^	90.00	Ge—O^xx^—O^xv^	45.00
O—Ge—O^xiii^	90.00	Ge—O^xx^—O^xvi^	45.00
O—Ge—O^xiv^	180.00	O—O^i^—O^ii^	60.00
O—Ge^i^—O^ii^	90.00	O—O^i^—O^xvi^	180.00
O—Ge^i^—O^xii^	90.00	O—O^i^—O^xii^	60.00
O—Ge^ii^—O^xiii^	90.00	O—O^ii^—O^xv^	180.00
Sr—O—Sr^xvii^	90.00	O—O^ii^—O^xiii^	60.00
Sr—O—Sr^xviii^	90.00	O—O^v^—O^vi^	60.00
Sr—O—Sr^xix^	180.00	O—O^v^—O^xv^	60.00
Sr—O—Ge	90.00	O—O^v^—O^xiii^	180.00
Sr—O—Ge^xx^	90.00	O—O^vi^—O^xvi^	60.00
Sr—O—O^i^	60.00	O—O^vi^—O^xii^	180.00
Sr—O—O^ii^	60.00	O—O^xv^—O^xvi^	60.00
Sr—O—O^v^	60.00	O—O^xii^—O^xiii^	60.00

Symmetry codes: (i) *z*, *x*, *y*; (ii) *y*, *z*, *x*; (iii) *y*−1, *z*−1, *x*; (iv) *z*−1, *x*, *y*−1; (v) *z*−1, *x*, *y*; (vi) *y*−1, *z*, *x*; (vii) *x*, *y*−1, *z*−1; (viii) *x*, *y*−1, *z*; (ix) *y*, *z*−1, *x*; (x) *x*, *y*, *z*−1; (xi) *z*, *x*, *y*−1; (xii) *y*, *z*, *x*+1; (xiii) *z*, *x*+1, *y*; (xiv) *x*+1, *y*, *z*; (xv) *y*−1, *z*, *x*+1; (xvi) *z*−1, *x*+1, *y*; (xvii) *x*, *y*, *z*+1; (xviii) *x*, *y*+1, *z*; (xix) *x*, *y*+1, *z*+1; (xx) *x*−1, *y*, *z*.
